# Case Report: A typical, nearly missed jejunal gallstone ileus

**DOI:** 10.3389/fgstr.2026.1870893

**Published:** 2026-08-18

**Authors:** Chengyang Chen, Yushi Fan, Baoping Tian, Hui Shan, Nanxia Xuan, Linlin Du, Gensheng Zhang

**Affiliations:** 1 Department of Critical Care Medicine, Second Affiliated Hospital, Zhejiang University School of Medicine, Hangzhou, China; 2 Department of Surgical Intensive Care Medicine, Second Affiliated Hospital, Zhejiang University School of Medicine, Hangzhou, China; 3 Key Laboratory of Multiple Organ Failure (Zhejiang University), Ministry of Education, Hangzhou, China; 4 Department of Critical Care Medicine, Taizhou Hospital of Zhejiang Province affiliated with Wenzhou Medical University, Taizhou, China

**Keywords:** cholecystoduodenal fistula, computed tomography, jejunal gallstone ileus, Rigler’s triad, rim-calcification

## Abstract

**Background:**

Gallstone ileus is a rare cause of mechanical intestinal obstruction, predominantly affecting older adults. The ileum and ileocecal valve are the most common sites of impaction, whereas jejunal involvement is considerably less frequent. Contrast-enhanced computed tomography (CT) is the primary imaging modality for diagnosis; however, minimally calcified ectopic stones are prone to being overlooked, leading to delayed surgical intervention.

**Case presentation:**

A 68-year-old woman presented with a 12-day history of abdominal pain, nausea, vomiting, and obstipation. She underwent two contrast-enhanced abdominal CT scans at different institutions, both of which demonstrated cholelithiasis, pneumobilia, and small bowel dilation, but failed to definitively identify an obstructive gallstone. Nevertheless, the combination of pneumobilia and small bowel obstruction—both components of Rigler’s triad—along with a visible cholecystoduodenal fistula, made gallstone ileus highly suspected. Emergency exploratory laparotomy revealed a 3.0 × 2.5 cm rim-calcified stone lodged in the jejunum, 80 cm distal to the ligament of Treitz. Simple enterolithotomy was performed without fistula repair. The patient recovered gradually, though she developed transient postoperative atrial fibrillation with hypotension on Day 21, which was managed medically. She was discharged on Day 30. Retrospective re-evaluation of the CT images confirmed the subtle rim-calcified stone.

**Conclusions:**

Minimally rim-calcified stones are easily missed on contrast-enhanced CT. In patients with intestinal obstruction exhibiting two of the three signs of Rigler’s triad, gallstone ileus should be strongly suspected even when no ectopic stone is clearly visible. Proactive clinician–radiologist communication and structured follow-up planning are essential to reduce diagnostic errors and manage recurrence risk.

## Introduction

Gallstone ileus is a rare form of mechanical intestinal obstruction, accounting for less than 0.1% of all causes of intestinal obstruction, and is more prevalent among older adults (1). It typically occurs when a gallstone enters the small intestine via a cholecystoduodenal fistula and subsequently causes an obstruction. The most common site of obstruction is the terminal ileum or ileocecal valve, with jejunal involvement being relatively uncommon.

Due to the non-specific nature of symptoms such as abdominal pain, nausea, vomiting, and abdominal distention, coupled with the fact that some gallstones are poorly visualized (radiolucent) on imaging, early or rapid diagnosis is often challenging. This frequently leads to delays in effective treatment and is associated with considerable mortality. We herein describe a typical case of jejunal gallstone ileus in an older woman. However, the diagnosis was nevertheless nearly missed, even after two contrast-enhanced abdominal CT scans, owing to the minimal calcification of the ectopic gallstone.

## Case presentation

A 68-year-old female patient presented to our emergency department with a 12-day history of abdominal pain accompanied by nausea, vomiting, and cessation of flatus and defecation. Ten days after symptom onset (Day 10), she showed no improvement and visited another hospital for evaluation. That hospital visit occurred two days before her admission to our center. A contrast-enhanced abdominal CT scan performed at that outside hospital demonstrated cholelithiasis, pneumobilia, and dilated small bowel with air-fluid levels, confirming small bowel obstruction but failing to identify an obstructive lesion. An intestinal obstruction tube was placed. Gastrointestinal decompression and anti-inflammatory therapy were administered without improvement, and she was subsequently transferred to our hospital’s emergency department. Her medical history included cholelithiasis and knee osteoarthritis, with no prior surgical history. She also reported chronic constipation.

On admission, her vital signs were as follows: blood pressure 147/61 mmHg, heart rate 78 beats per minute, respiratory rate 20 breaths per minute, and peripheral oxygen saturation of 97% on room air; and no fever. Physical examination revealed abdominal distention, tenderness, and mild rebound tenderness localized to the right abdomen. Bowel sounds were active at eight times per minute. No obvious guarding, peritoneal irritation signs, or inguinal hernia were detected. Laboratory findings included leukocytosis and elevated C-reactive protein levels (**Table 1**).

**Table 1:** Blood laboratory findings on admission.

Laboratory test	Results	Reference range	Unit
RBC	4.75	3.68–5.13	×10^12^/L
Hb	137	113–151	g/L
NEUT	78.3%	50.0–70.0%	–
Plt	473	100–300	×10^9^/L
WBC	16.4	4.0–10.0	×10^9^/L
CRP	31.6	<10.0	mg/L
PCT	0.48	<0.5	ng/mL
IL-6	45.6	<7	pg/mL
Albumin	36.8	35.0–52.0	g/L
TBIL	18.6	<26.0	μmo1/L
DBIL	1.4	<5.0	μmo1/L
IBIL	17.2	<19.0	μmo1/L
ALT	20	9–50	U/L
AST	24	15–40	U/L
Cr	47.9	41.0–83.0	μmol/L
PT	12.8	12.0-14.0	s
APTT	45.7	30.0-45.0	s
Fbg	5.63	2.0-4.0	g/L

**Figure 1:**
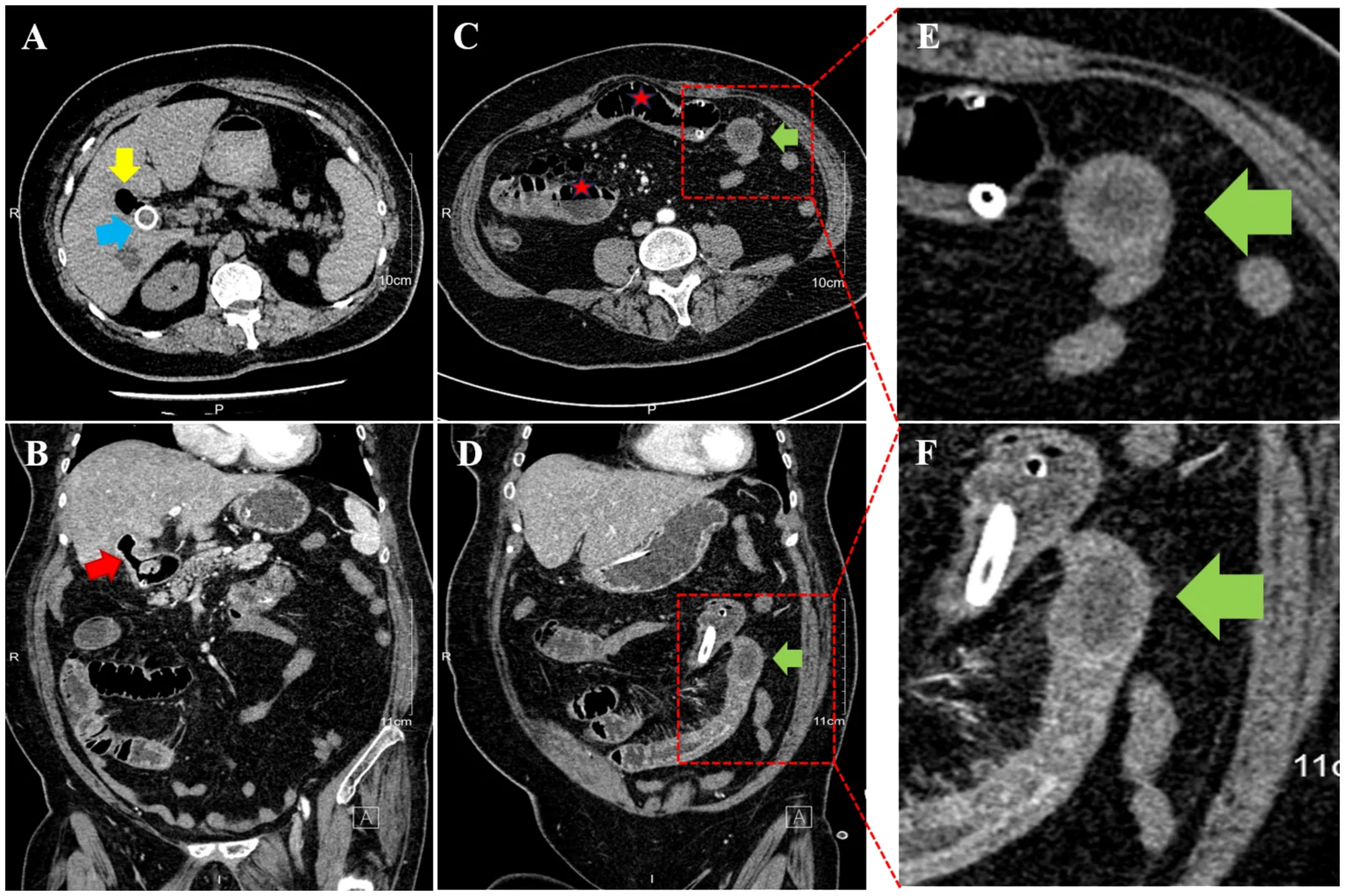
Abdominal CT scan in coronal and axial planes. **(A)** Pneumobilia with gas in the gallbladder (yellow arrow) and a gallstone with rim calcification (blue arrow). **(B)** Internal fistula between the gallbladder and duodenum (red arrow). **(C, D)** Contrast-enhanced CT showing a faintly rim-calcified gallstone within the jejunum (green arrow) and air-fluid levels in the bowel (pentagram). **(E, F)** Magnified images of the jejunal stone (green arrow).

The second contrast-enhanced CT scan revealed cholelithiasis, pneumobilia, a cholecystoduodenal fistula, left-sided small bowel wall edema, exudation, proximal jejunal dilation, multiple air-fluid levels, and ascites (**Figures 1A, B**). She presented with a clear diagnosis of acute small bowel obstruction. The absence of prior abdominal surgery and hernial signs effectively ruled out adhesion- and hernia-related causes, while CT showed no evidence of tumors or inflammatory bowel disease. However, CT clearly demonstrated cholelithiasis, pneumobilia, and a cholecystoduodenal fistula, providing a definitive anatomical pathway for gallstone migration into the intestinal tract. Combined with the patient’s age and sex, which closely matched the typical epidemiology of gallstone ileus, this diagnosis was prioritized preoperatively. She subsequently underwent an emergency exploratory laparotomy. Hypotension occurred after induction of anesthesia and was promptly corrected with vasoactive agents.

Intraoperative findings included a small amount of yellowish ascites and a firm, spherical mass within the jejunum, approximately 80 cm distal to the ligament of Treitz. The proximal bowel wall was edematous and thickened, whereas the distal bowel showed milder edema. The intestinal blood supply was preserved, with no evidence of ischemia or necrosis.

**Figure 2:**
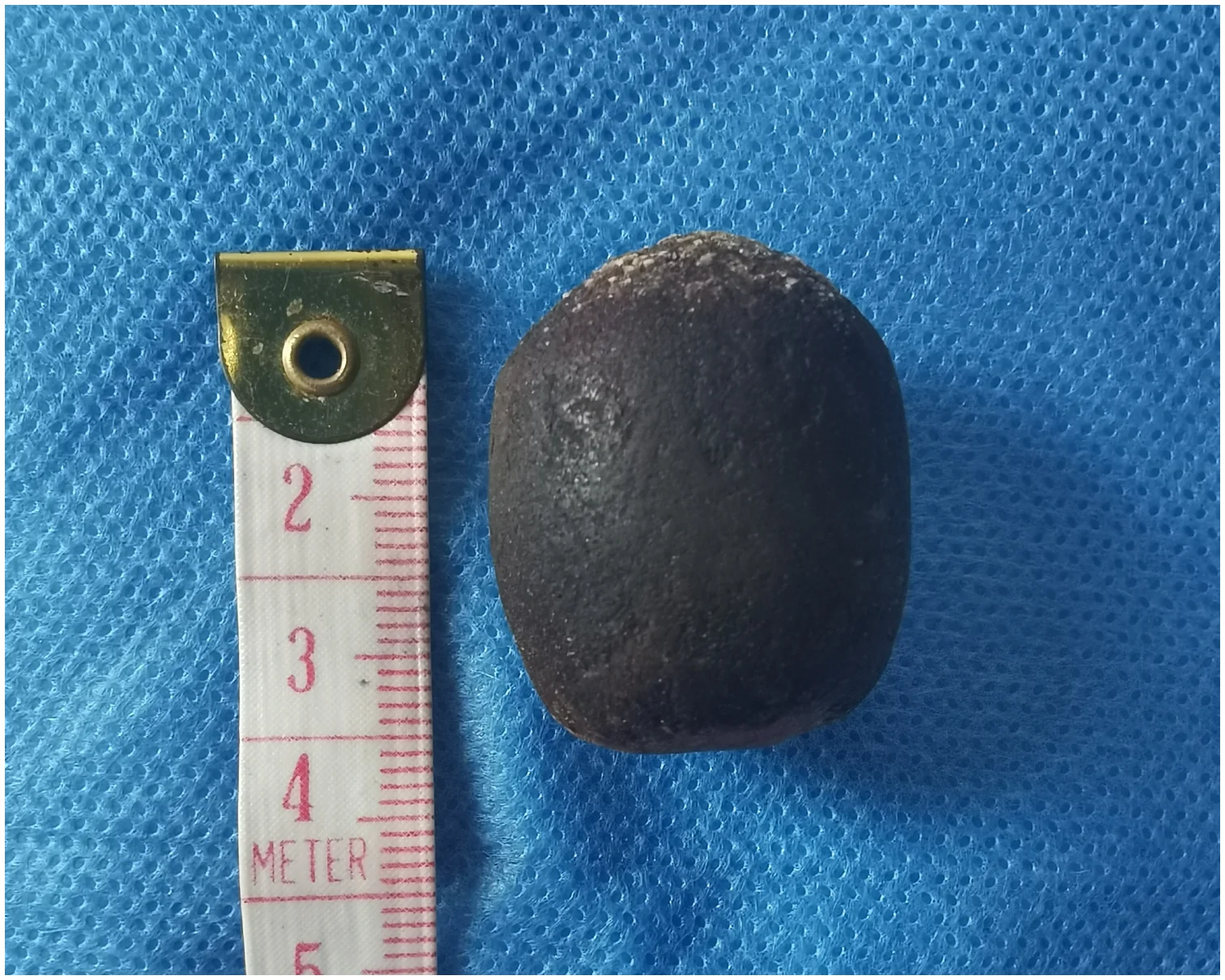
A dark brown gallstone was surgically removed from the jejunum via enterotomy.

Given the patient’s advanced age, prolonged duration of intestinal obstruction, intraoperative hypotension with shock, and substantial systemic inflammatory response, we decided to perform enterolithotomy alone without addressing the gallbladder or the cholecystoduodenal fistula to shorten operative time, minimize surgical risk, and reduce overall trauma. A dark brown gallstone measuring approximately 3 cm × 2.5 cm was completely removed from the jejunum (**Figure 2**).

**Figure 3:**
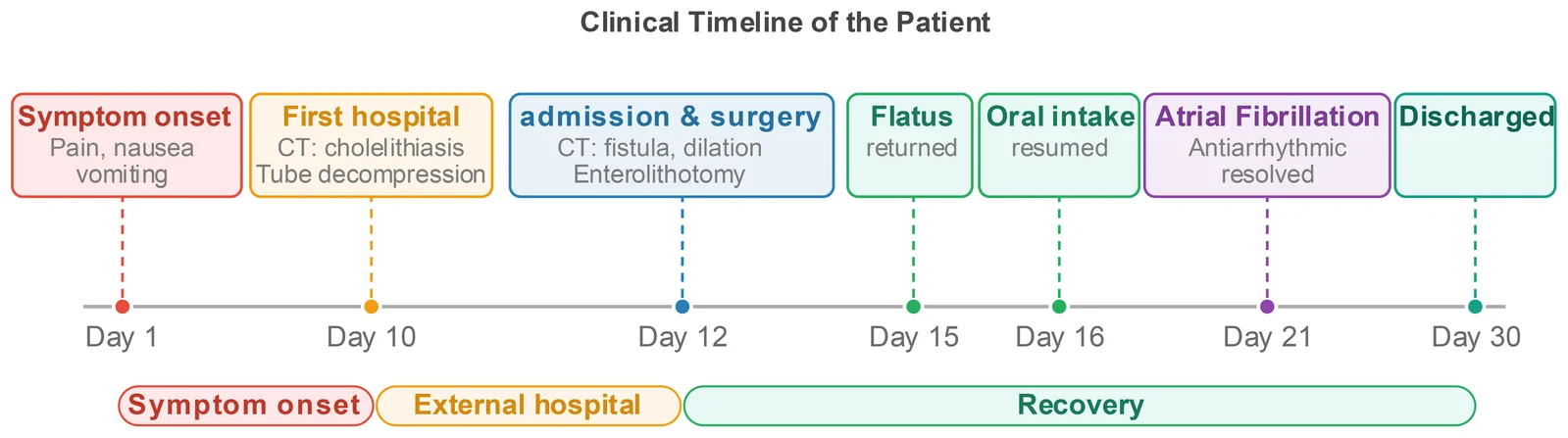
Clinical timeline of the patient.

Imipenem/cilastatin was administered perioperatively. Flatus returned on Day 15, and oral intake resumed on Day 16 after symptom onset. On Day 21, the patient developed rapid atrial fibrillation accompanied by hypotension, which improved after antiarrhythmic treatment. The incision healed well, and the patient was discharged on Day 30 after symptom onset (**Figure 3**). No follow-up data were available after discharge. Following postoperative communication with the radiologists, a retrospective review of the abdominal CT images was conducted. A subtle transition zone between dilated and collapsed small bowel segments was identified. This careful re-evaluation identified a minimally rim-calcified stone measuring approximately 3 cm × 2 cm within the jejunum (**Figures 1C–F**), which corresponded to the surgical findings.

## Discussion

Our patient exhibited classic radiographic signs of gallstone ileus on contrast-enhanced abdominal CT, including pneumobilia, intestinal obstruction, an ectopic jejunal stone, and the less commonly visualized cholecystoduodenal fistula (2). Together with her clinical symptoms of bowel obstruction, this constituted a typical, albeit relatively uncommon, presentation of jejunal gallstone ileus. However, the causative stone, characterized by subtle rim calcification, was initially missed by radiologists at two separate institutions, even after two separate enhanced CT scans. Despite this, the surgical team, integrating the key imaging findings (**Figure 1**) with the patient’s clinical presentation, strongly suspected gallstone ileus and proceeded with exploratory laparotomy, which successfully identified the stone. Following postoperative clinical feedback, a dedicated re-evaluation of the CT images by the radiologists ultimately revealed the subtly rim-calcified stone within the left-sided small bowel.

Gallstone ileus is a rare cause of mechanical intestinal obstruction, constituting less than 0.1% of all intestinal obstructions (1). However, it accounts for approximately one-quarter of non-strangulating intestinal obstructions in patients over 65 years of age. It has a predilection for women, with a female-to-male ratio of approximately 3.5:1 (3, 4). Indeed, our patient was a 68-year-old older woman. The diagnosis is frequently delayed because symptoms such as intermittent abdominal pain, nausea, and vomiting are non-specific and often indistinguishable from those of other causes of intestinal obstruction (5). Such delays in diagnosis and treatment are associated with the considerable mortality reported for this condition (6, 7). The initial diagnostic oversight in our case can partly be attributed to this lack of specific symptomatology.

In the majority of cases (75%–83%), gallstones enter the duodenum through a cholecystoduodenal fistula (8). This internal fistula forms due to vascular insufficiency caused by pressure erosion from the stone or chronic inflammation, leading to communication between the gallbladder and duodenal walls. According to the study by Yu et al. (2), direct visualization of a cholecystoduodenal fistula on CT is uncommon, occurring in only approximately 21% of patients with gallstone ileus. Our patient exhibited this less frequent finding on her enhanced CT. Once within the intestinal tract, the gallstone may impact at any site. Reported data indicate the most common site of impaction is the ileum or ileocecal valve (50%–60.5%), with jejunal involvement being less frequent (16.1%–26.9%) (9). Our patient represents one of these less common cases of jejunal gallstone ileus. The relative rarity of jejunal obstruction may have contributed to the initial oversight by both radiologists.

The classic radiographic triad for gallstone ileus—pneumobilia, ectopic gallstone, and intestinal obstruction—is known as Rigler’s triad (10, 11). The presence of any two of these three signs is sufficient to suggest the diagnosis, even in the absence of a definitively identified ectopic stone on imaging (12, 13). While the complete triad is found in fewer than 50% of patients on plain abdominal radiography, its detection rate is significantly higher on contrast-enhanced abdominal CT, which can substantially reduce the rate of missed diagnoses (1, 12, 14). In a retrospective analysis evaluating the utility of CT for diagnosing gallstone ileus, Yu et al. (2) reported a sensitivity of 93%, a specificity of 100%, and an accuracy of 99%, with 13 of 14 patients exhibiting the complete Rigler’s triad. Our patient presented a classic example, exhibiting the full Rigler’s triad. Had the radiologists recognized that the patient already displayed two key components of the triad (pneumobilia and intestinal obstruction), a diagnosis of gallstone ileus could have been strongly considered even without definitive visualization of an ectopic stone. The initial miss, therefore, also points to a potential gap in awareness of the diagnostic criteria for this condition among interpreting radiologists.

While CT typically reveals the size and location of an obstructing stone, not all gallstones are clearly visualized (13). Identifying rim-calcified stones on contrast-enhanced CT is more challenging than identifying densely calcified ones. Consequently, rim-calcified stones are more easily missed, particularly when calcification is minimal, resulting in poor attenuation difference from the enhanced bowel wall, and when the stone is closely apposed to the wall, further obscuring its margins. This scenario, however, appears to be uncommon. In the series analyzed by Yu et al. (2), only one of 14 cases was a CT false-negative due to a minimally rim-calcified stone that was missed by two radiologists. Our case is similar: the ectopic stone had subtle rim calcification, rendering its density similar to that of the enhanced intestinal wall, with poor contrast and no clear plane of separation; the faint rim could easily be mistaken for the bowel wall itself. We suggest that this was the primary reason for the radiographic oversight. Additionally, the presence of a densely calcified stone within the gallbladder on the same CT scan might have inadvertently biased the radiologists toward expecting clearly calcified stones, potentially diverting attention from the poorly visualized obstructive stone.

The stone was identified on the CT images only after postoperative communication between the surgeons and radiologists. Had the surgeons shared their diagnostic reasoning and suspicion with the radiology team preoperatively, the exact location of the obstruction might have been identified earlier. This could have led to a more precise surgical plan and potentially improved the patient’s outcome. We therefore emphasize that, in cases of intestinal obstruction where clinical findings and imaging appearances are discordant, proactive interdisciplinary communication and formal multidisciplinary case discussion are crucial to minimize the likelihood of diagnostic error.

Conservative management of gallstone ileus has a low success rate, and the majority of patients require surgical intervention. The primary therapeutic goals are stone extraction and relief of intestinal obstruction (15). Three main surgical approaches are available: simple enterolithotomy alone; one-stage enterolithotomy with concurrent cholecystectomy and fistula repair; and a two-stage strategy consisting of initial enterolithotomy followed by elective cholecystectomy and fistula repair 4–6 weeks later. Simple enterolithotomy is the most commonly used approach, offering less trauma and lower surgical risk, but it is associated with a 5%–9% recurrence rate due to the untreated biliary-enteric fistula. The one-stage procedure carries greater trauma and higher perioperative risk, though it completely eliminates recurrence. The choice of surgical strategy should be individualized based on the patient’s overall condition and comorbidities (16). Notably, the patient developed a transient episode of rapid atrial fibrillation with hypotension on postoperative Day 21. This complication was likely multifactorial in origin, attributable to the sustained systemic inflammatory response, prolonged critical illness, and surgical stress, although an underlying subclinical cardiac predisposition could not be entirely excluded. This event underscores the importance of vigilant postoperative cardiovascular monitoring in older patients with prolonged gallstone ileus, as cardiac complications may significantly influence recovery and discharge planning.

This case report has several limitations. First, as a single-case retrospective report, it cannot provide statistically generalizable conclusions. Second, the causative stone was identified only upon retrospective image review in consultation with radiologists postoperatively. This “*post-hoc* identification” limits the strength of our conclusions regarding preoperative imaging diagnostic strategies. Third, no outpatient follow-up data were available after discharge, leaving information on long-term recovery, potential stone recurrence, or biliary complications missing, which somewhat limits the value of this report in assessing long-term prognosis. Nevertheless, the diagnostic dilemma and clinical decision-making process presented in this case offer educational value and serve as a useful clinical reference for practicing physicians.

## Conclusion

Gallstone ileus is a rare cause of mechanical intestinal obstruction. Obstruction at the jejunal level is uncommon compared with ileal obstruction. Contrast-enhanced abdominal CT is a key imaging technique for diagnosing gallstone ileus; however, identifying minimally rim-calcified stones remains challenging, and such stones are easily overlooked. In cases of intestinal obstruction of uncertain etiology, enhanced communication between clinicians and radiologists, along with multidisciplinary case discussions, should be pursued to reduce the likelihood of missed diagnoses. In addition, given that the cholecystoduodenal fistula was left *in situ* after simple enterolithotomy, patients should be explicitly counseled regarding the 5%–9% risk of recurrence and offered elective follow-up planning to enable early detection and management of potential biliary complications.

## Data Availability

The original contributions presented in the study are included in the article/supplementary material. Further inquiries can be directed to the corresponding author.
